# Retraction Note: ROCK1 mechano-signaling dependency of human malignancies driven by TEAD/YAP activation

**DOI:** 10.1038/s41467-025-65053-y

**Published:** 2025-10-20

**Authors:** Davide Esposito, Ila Pant, Yao Shen, Rui F. Qiao, Xiaobao Yang, Yiyang Bai, Jian Jin, Poulikos I. Poulikakos, Stuart A. Aaronson

**Affiliations:** 1 Department of Oncological Sciences, Tisch Cancer Institute, Icahn School of Medicine at Mount Sinai, New York, NY 10029 USA; 2 Mount Sinai Center for Therapeutics Discovery, Departments of Pharmacological Sciences and Oncological Sciences, Tisch Cancer Institute, Icahn School of Medicine at Mount Sinai, New York, NY 10029 USA; 3 Department of Medical Oncology, The First Affiliated Hospital of Xi’an Jiaotong University, Xi’an, 710061 China; 4 Department of Dermatology, The Tisch Cancer Institute, Icahn School of Medicine at Mount Sinai, New York, NY 10029 USA

Retraction to: *Nature Communications* 10.1038/s41467-022-28319-3, published online 4 February 2022

The Editors have retracted this Article.

After publication, concerns were raised regarding the images presented in this article, specifically:

1. Fig 1c p53 R273H-EV and p53 R175H-EV appear similar after horizontal flip.

2. Fig 1c YAP WT-EV and Fig 2j p53 R273H -DMSO appear similar.

3. Fig 1f p53 DNA contact mutant cells - HCC193-Ctr and Fig 2I p53 DNA contact mutant cells - HCC193-DMSO appear similar.

4. Fig 1f p53 null cells-HCC1806-dhTEAD4 and Supp Fig 3d p53 conformational mutant cells-HCC1395-DMSO appear similar.

5. Fig 1f p53 null cells-HC1806-shp53 and Supp fig 3d - p53 conformational mutant cells -HCC1395-GGTI-298 appear similar.

6. Fig 1f p53 null cells - HCC1806-Ctr and Supp Fig 3d - p53 conformational mutant cells - HCC1395-GGTI-298 appear similar.

7. Supp Fig 7 p53 DNA mutant cellls - HCC193 (H-1152 and Simvast) appear similar.

Authors were unable to provide the original raw data for the images for which concerns were raised. The Editors therefore no longer have confidence in the research presented in this work.

Stuart A. Aaronson disagrees with this retraction. None of the remaining authors have responded to correspondence from the Publisher about this retraction.

